# P330: Translating regional patient safety and infection prevention mandates into local action in african hospitals: the power of context specific improvement resources

**DOI:** 10.1186/2047-2994-2-S1-P330

**Published:** 2013-06-20

**Authors:** J Storr, S Syed, J Hightower, R Gooden, S Bagheri Nejad

**Affiliations:** 1 WHO patient safety, WHO, Geneva, Switzerland; 2 WHO office, WHO, Harare, Zimbabwe

## Introduction

At the 58^th^ WHO African Regional Committee all 46 countries endorsed a paper on patient safety calling for action in 12 areas. Infection prevention and control (IPC) was highlighted as a cornerstone action area. The paper acted as a catalyst for kinetic action that is now translating a regional mandate into positive changes to hospital safety and IPC across Africa, focusing on a triad of usable IPC improvement resources.

## Objectives

To develop three key patient safety resources for hospitals in Africa.

## Methods

A literature review on quality improvement drove the development of key resources, informed by a mapping of the regional mandate, framed around the 12 action areas, against the local contexts of African Partnerships for Patient Safety hospitals established through field visits.

## Results

Over 50 papers were reviewed and six hospitals visited. Three key resources were co-developed 1) a situational analysis to enable establishment of the state of IPC and patient safety in a healthcare facility 2) an improvement framework to prepare hospitals for action on IPC and patient safety 3) a resource map to inform improvement and assist with implementation and sustainability.

## Conclusion

The Situational Analysis allows for rapid collection of information. The Partnership Preparation Pack outlines an improvement approach and lists key considerations for improvers at the start of their journey. The APPS Resource Map spans guidance, policies, publications and toolkits. This triad is the first of a kind package for patient safety improvement in the African Region with IPC at its heart. Lessons from early implementers demonstrate its utility in initiating small-scale change with potential to result in system-wide and in-country improvement. Alignment of IPC improvement resources with policy has been central to creating impetus and action on IPC and patient safety across partnership hospitals, and strengthens the translation of mandate to action. A unique feature has been direct involvement of front line health-care workers in shaping the resources. The approach illustrates the impact that global thought leadership can have when it interacts with front line workers to co-develop context specific resources for action.

## Disclosure of interest

None declared

